# Intervention in the Timeliness of Two Electrocardiography Types for Patients in the Emergency Department With Chest Pain: Randomized Controlled Trial

**DOI:** 10.2196/36335

**Published:** 2022-09-13

**Authors:** Suyoung Yoo, Hansol Chang, Taerim kim, Hee yoon, Sung Yeon Hwang, Tae Gun Shin, Min Seob Sim, Ik joon Jo, Jin-Ho Choi, Won Chul Cha

**Affiliations:** 1 Department of Digital Health Samsung Advanced Institute for Health Science & Technology Sungkyunkwan University Seoul Republic of Korea; 2 Department of Emergency Medicine Samsung Medical Center Sungkyunkwan University School of Medicine Seoul Republic of Korea; 3 Health Information and Strategy Center Samsung Medical Center Seoul Republic of Korea

**Keywords:** imaging, electrocardiography, wireless technology, emergency department, emergency, angina, ECG, EKG, cardiology, chest, pain, electrocardiogram, randomized, randomization, heart, cardiac, diagnose, diagnosis, accuracy

## Abstract

**Background:**

In the emergency department (ED), the result obtained using the 12-lead electrocardiography (ECG) is the basis for diagnosing and treating patients with chest pain. It was found that performing ECG at the appropriate time could improve treatment outcomes. Hence, a wearable ECG device with a timer can ensure that the findings are continuously recorded.

**Objective:**

We aimed to compare the time accuracy of a single-patch 12-lead ECG (SP-ECG) with that of conventional ECG (C-ECG). We hypothesized that SP-ECG would result in better time accuracy.

**Methods:**

Adult patients who visited the emergency room with chest pain but were not in shock were randomly assigned to one of the following 2 groups: the SP-ECG group or the C-ECG group. The final analysis included 33 (92%) of the 36 patients recruited. The primary outcome was the comparison of the time taken by the 2 groups to record the ECG. The average ages of the participants in the SP-ECG and C-ECG groups were 63.7 (SD 18.4) and 58.1 (SD 12.4) years, respectively.

**Results:**

With a power of 0.95 and effect sizes of 0.05 and 1.36, the minimum number of samples was calculated. The minimum sample size for each SP-ECG and C-ECG group is 15.36 participants, assuming a 20% dropout rate. As a result, 36 patients with chest pain participated, and 33 of them were analyzed. The timeliness of SP-ECG and C-ECG for the first follow-up ECG was 87.5% and 47.0%, respectively (*P*=.74). It was 75.0% and 35.2% at the second follow-up, respectively (*P*=.71).

**Conclusions:**

Continuous ECG monitoring with minimal interference from other examinations is feasible and essential in complex ED situations. However, the precision of SP-ECG has not yet been proved. Nevertheless, the application of SP-ECG is expected to improve overcrowding and human resource shortages in EDs, though more research is needed.

**Trial Registration:**

ClinicalTrials.gov NCT04114760; https://clinicaltrials.gov/ct2/show/NCT04114760

## Introduction

Twelve-lead electrocardiography (ECG) is an essential diagnostic tool in the emergency department (ED) for patients with chest pain [1]. The most important step to be taken for a patient who complains of chest pain is to identify the location of the pain. ECG should be performed to determine if the pain is caused by a cardiovascular disease [2]. According to the guideline of the Journal of European Heart, ECG was performed as the first step for evaluation and treatment when patients with chest pain visited the ED [3]. Currently, ED in South Korea is following the American Heart Association’s recommendation to take an initial ECG within 10 minutes of the patient’s visit with chest pain [4]. This is to determine whether it is chest pain caused by coronary artery disease and to improve the patient’s clinical results through quick treatment if necessary. This is because rapid diagnosis of ST elevation myocardial infraction, or acute myocardial infarction, requires immediate treatment, and treatment among coronary artery diseases leads to a decrease in the patient’s prognosis or mortality [5]. From 8.5% to 40% of patients with acute myocardial infarction have symptoms; however, the rise of the ST segment, which determines whether to perform an immediate procedure, is not visible on the ECG, and then the rise of the ST segment occurs over time [6].

Therefore, continuous ECG monitoring after the initial ECG is important [7]. If ECG is not performed on time, this may affect the clinical outcome of the patient. Timely ECG is associated with improved clinical outcomes in patients with serious cardiovascular diseases. Electrocardiographic findings during acute myocardial infarction can vary substantially depending on the type, stage, and extent of infarction and timing of the ECG acquisition [8-10]. Therefore, a delayed ECG in the ED, which can be due to overcrowding or shortages in human resources, can result in poor patient outcomes [11-13].

Complex and unstable circumstances in the ED are challenging for current monitoring systems. Patients often move from one location to another for various tests and procedures. In addition, the long physical lines and multiple large patches of current ECG devices are not suitable for long-term use and are time-consuming. Hence, a single-patch wireless 12-lead ECG (SP-ECG) with a timer can be beneficial in cases of predecided follow-up ECG. A single patch would enable patients to move outside the bed and could be used in complex emergency room situations requiring many tests. However, to the best of our knowledge, no studies have been conducted on such devices in ED settings. Thus, this study aimed to evaluate the effect of SP-ECG with a timer on the timeliness of follow-up ECG in the clinical setting for patients with chest pain.

## Methods

### Study Design

This was a prospective randomized controlled study conducted in the ED of an academic tertiary hospital. Participants who visited ED with chest pain were randomly assigned into the 2 groups of conventional ECG (C-ECG) and single-patch 12-lead ECG (SP-ECG). The main comparison variable was the timeliness of the recording time for the 2 ECG types. The study protocol was registered at ClinicalTrials.gov (NCT04114760). To clarify the methods, we followed CONSORT (Consolidated Standards of Reporting Trials) checklist (Multimedia Appendix 1) [14].

### Study Setting

This study was conducted in the ED of an academic tertiary hospital in Seoul with approximately 2000 inpatient beds and around 2,000,000 annual outpatient visits. The average number of ED admissions is 78,000 per year. The first study participants were enrolled on July 30, 2020, while the last study participants were enrolled on October 8, 2020. The study was conducted for approximately 70 days.

### Recruitment

Patients who visited the hospital’s ED with chest pain as the chief complaint were considered for inclusion in the study. The inclusion criteria were as follows: visits to the ED with chest pain or chest discomfort as the chief complaint, age more than 19 years, the ability to stay in the emergency room during the study, and provision of consent to participate in the study. The exclusion criteria were as follows: refusal to provide consent to participate, active “Do Not Resuscitate” order, shock or cardiac arrest status, Patients with Korean Triage and Acuity Scale score 2 to 5, and ST-segment elevation myocardial infarction observed in the first ECG test.

### Study Protocol

**Figure 1:**
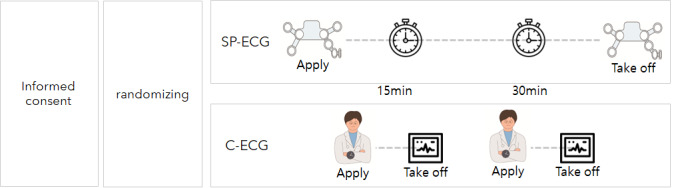
Study protocol. C-ECG: conventional electrocardiography (ECG); SP-ECG: single-patch 12-lead ECG.

All patients with a chief complaint of chest pain were examined using an ECG immediately after visiting the ED and were randomly divided into the SP-ECG and C-ECG groups after the confirmation of the absence of ST-segment elevation myocardial infarction and shock [15]. Both groups were required to undergo ECG measurements twice every 15 minutes from the baseline [16]. The criterion for the SP-ECG group was the time set on the device, whereas that for the control group was the order time of the physician after the patient was assigned a bed (Figure 1).

### Device and System

**Figure 2:**
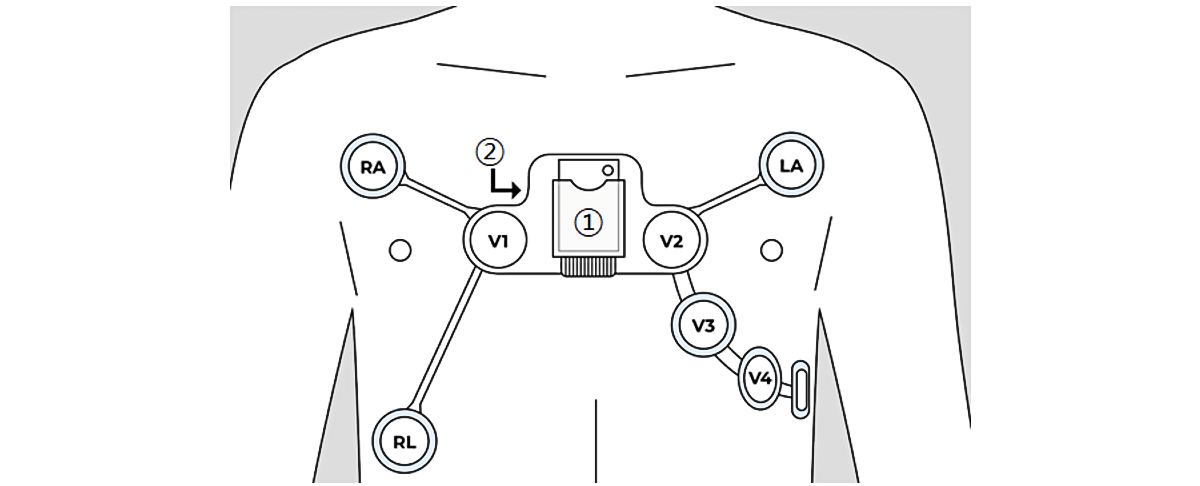
Design configuration. 1. Main socket: socket of the patch for performing 12-lead electrocardiography. 2. Single-patch–type electrode. LA: left arm; RA: right arm; RL: right leg; V1-V4: voltage1-voltage.

The devices used in the study were a page writer TC70 (existing device) and Healthrian SP-ECG. Healthrian SP-ECG was used as an intervention device and was approved by the Korean Ministry of Food and Drug Administration as a Holter ECG monitor (Figure 2).

Figure 2 shows the design configuration, where the main socket and the single-patch–type electrode are located. It consists of a main body and single-patch–type electrode. The dimensions of the main body were 46 × 35.6 × 16 millimeters with a weight of 30 grams. The patch measured 241.19 × 375.5 millimeters and weighed 35 grams. The main body was assembled in the socket of the patch. To perform the 12-lead ECG examination, the tablet and main body were wirelessly connected via Bluetooth for continuous monitoring.

**Figure 3:**
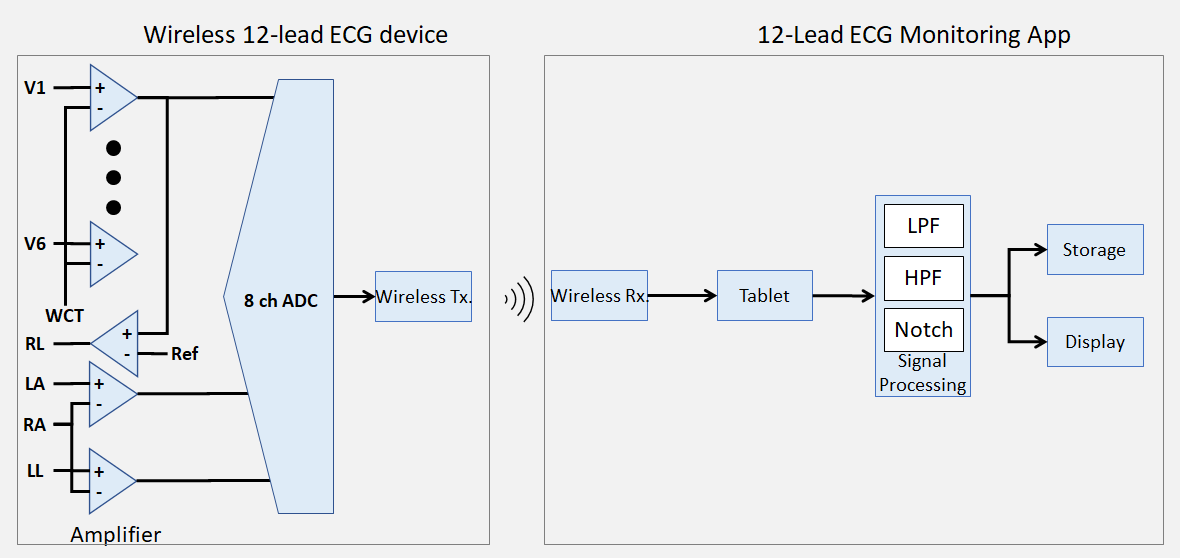
System architecture of the single-patch 12-lead electrocardiography (ECG). 8 ch DC: converts an amplified analog signal into a digital signal;amplifier: amplifies analog voltage obtained from the electrodes; digital signal processing: computes received digital signals as ECG signals through digital operations; display: converts output processed ECG data into a visualization graph; HPF (high-pass filter): eliminates low-frequency noise; LA: left arm; LL: left leg; LPF (low-pass filter): eliminates high-frequency noise; notch (notch filter): eliminates noise at a certain frequency, eliminates 60-Hz noise used for commercial power sources; RA: right arm; RL: right leg; storage: stores processed ECG data; V1-V4: voltage1-voltage4; WCT: Wilson’s Central Terminal.

Figure 3 shows the system architecture of SP-ECG. The main board of the device is based on a context-m4 digital signal-processing board. The analog front end consists of an ECG amplifier and an analog-digital converter to obtain a signal [17]. The ECG results were generated in a portable document format and were transmitted in real time to the researcher’s dashboard via Long-Term Evolution networks when the tablet’s “upload” button was clicked. The tablet used in this study was a Samsung Galaxy Tab S3 (SM-T825), which was connected to an LTE network. The dashboard used in this study was the Samsung Galaxy book (SM-W627NZFKOO), which could also be used on a PC. In addition, it used an LTE network. The differences between C-ECG and SP-ECG are significant. C-ECG has 10 separate electrodes, each connected to the patient by a physical long line. In SP-ECG, a single patch–type ECG is used to measure an ECG wirelessly. A signal is picked up and sent from a socket inside the single patch–type ECG.

### Measurement

The primary outcome in this study was the timeliness of the ECG measurements. The study protocol required subjects in both groups to be subjected to an ECG twice at 15-minute intervals. Participants in the C-ECG group underwent manual measurements by medical personnel at a specified time using the same 12-lead ECG. In the SP-ECG group, each ECG was automatically generated at a specified time using a pre-attached device. The SP-ECG device was attached to the participants at study initiation (time 0) and was removed after 1 hour.

Follow-up ECG was considered timely when it was taken within 3 minutes of the prespecified time. For example, if an ECG was recorded 14 minutes after the initial ECG, it was considered timely. Covariates, such as age, sex, Korean Triage and Acuity Scale score, heart rate, body temperature, respiratory rate, and blood pressure, were recorded based on the subjects’ initial triage information at the ED.

### Statistical Analysis

All data were stored in a Microsoft Excel spreadsheet. *P* values compared to baseline characteristics and time differences were calculated by comparing the means using the chi-square test. We compared the time differences between the 2 groups by comparing the means and standard deviations. Statistical significance was set at *P*<.05.

### Sample Size

The *P* values in relation to the baseline characteristics and time differences were calculated by comparing the means via the chi-square test, 2-sample 2-tailed *t* tests, and statistical tests. The minimum number of samples for verification of the experimental hypothesis was calculated with a power of 0.95 and effect sizes of 0.05 and 1.36.

Based on previous pilot studies, 15 was calculated as the minimum sample size for each test and control group. Assuming a dropout rate of 20%, the total number of participants was 36.

### Ethics Approval

The protocol of this study was reviewed and approved by Samsung Hospital’s Institutional Review Board (IRB #2019-01-046-008).

## Results

A total of 36 participants were enrolled in this study. The median ages in the SP-ECG and C-ECG groups were 63.7 (SD 18.4) and 58.1 (SD 12.4), respectively. Of the 36 enrolled patients, 33 (92%) were included in this study. One of the excluded patients wanted to drop out of the study due to disorientation. In one participant from each group, there were errors in the time measurements due to study violation. The average age of the 33 final participants was 61.06 (SD 15.8) years. Moreover, 14/33 (42.4%) patients were women, and the most common Korean Triage and Acuity Scale was 3. The other characteristics did not show significant intergroup differences (Table 1).

Intergroup differences in age, sex, and vital signs were small. Although 12/17 (70%) of the C-ECG group had a Korean Triage and Acuity Scale of 3, this was not meaningful because the participants were randomly assigned to each group.

**Table 1:** Demographic information of the study participants.

Characteristics		C-ECG^a^ group (n=17)	SP-ECG^b^ group (n=16)	*P* value	
Age (years), mean (SD)		63.7 (18.4)	58.1 (12.5)	.32	
**Sex, n (%)**					
	Female	6 (35)	8 (50)		
	Male, N	11 (65)	8 (50)		
**KTAS^c^, n (%)**					.80
	1	0	0		
	2	3 (18)	6 (38)		
	3	12 (70)	5 (31)		
	4	2 (12)	5 (31)		
	5	0	0		
Heart rate (beats per minute), median (SD)		79.4 (17.9)	79.3 (12.6)	.99	
Body temperature (°C), median (SD)		36.7 (0.5)	36.7 (0.4)	.82	
Respiratory rate (breaths per minute), median (SD)		18.5 (2.7)	18.1 (1.7)	.61	
Systolic blood pressure (mmHg), median (SD)		133.4 (18.4)	129.8 (18.2)	.57	
Diastolic blood pressure (mmHg), median (SD)		79.7 (14.3)	81.3 (14.6)	.75	

^a^C-ECG: conventional electrocardiography.

^b^SP-ECG: single-patch 12-lead ECG.

^c^KTAS: Korean Triage and Acuity Scale.

### Main Outcome

#### Timeliness of the ECG Types

**Figure 4:**
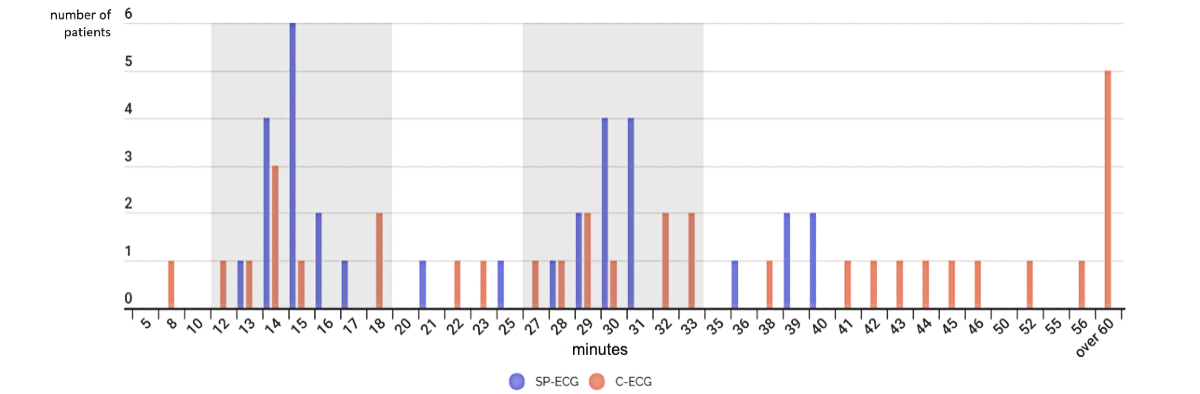
Timing of electrocardiography measurements (gray areas indicate accurate time intervals). C-ECG: conventional electrocardiography (ECG); SP-ECG: single-patch 12-lead ECG.

Figure 4 shows the timing distribution of the 2 study groups. The average times for the C-ECG group are 23 minutes and 68 minutes for each test, and the average times for the SP-ECG group are 15 minutes and 32 minutes for each test. The recorded ECG time for each patient assigned to the C-ECG group and SP-ECG group can be seen in Table 2.

For the first follow-up ECG, the timeliness values of the recordings in the SP-ECG and C-ECG groups were 13/16 (81%) patients and 7/17 (41%) patients, respectively (*P*=.74). At the second follow-up, it was 10/16 (63%) patients and 6/17 (35%) patients, respectively (*P*=.71). Overall, the accuracies were 81.2% and 41.1%, respectively (*P*=.62).

**Table 2:** Recorded electrocardiography (ECG) time for each patient assigned to the conventional ECG (C-ECG) group and the single-patch 12-lead ECG (SP-ECG) group.

C-ECG group			SP-ECG group		
Patient	1st follow-up ECG	2nd follow-up ECG	Patient	1st follow-up ECG	2nd follow-up ECG
S01	23	45	S02	15	30
S06	42	56	S03	15	31
S09	8	27	S04	15	31
S10	14	32	S08	29	44
S14	12	28	S11	16	30
S15	39	61	S12	17	32
S16	13	52	S13	15	39
S17	29	43	S18	14	40
S20	18	33	S19	15	31
S22	29	75	S21	14	31
S25	44	266	S23	14	28
S26	14	32	S24	15	30
S27	14	33	S28	15	30
S29	15	46	S30	16	29
S31	22	180	S32	20	35
S33	18	18	S36	16	31
S35	30	131	N/A^a^	N/A	N/A

^a^N/A: not applicable.

Figure 4 also illustrates that the C-ECG group’s timing is not only outside the targeted time window, but it is significantly more delayed than that of the C-ECG group. It is also noteworthy that the ECG was recorded for 4 participants from the C-ECG group more than one hour after Time 0.

The C-ECG group consisted of 6/17 (35%) women and 11/17 (65%) men, whereas the SP-ECG group consisted of 8/16 (50%) women and 8/16 (50%) men. In the C-ECG group, the average time of the first follow-up ECG in women (n=6) was 22 (SD 6.35) minutes, while that of the second follow-up ECG was 82.5 (SD 55.20) minutes. For men (n=11), the average time of the first follow-up ECG was 22 (SD 12.55) minutes, while that of the second ECG was 60 (SD 66.28) minutes. In the SP-ECG group, the average time for women’s (n=8) first follow-up ECG was 15 (SD 1) minutes, while that of the second ECG was 32 (SD 3.98) minutes. For men in the SP-ECG group (n=8), the average time for the first follow-up ECG was 17 (SD 4.58) minutes, while that for the second follow-up ECG was 32 (SD 4.71) minutes.

## Discussion

### Principal Findings

Wireless and single-patch ECGs were obtained from patients with chest pain at a more accurate time than C-ECG in clinical settings. Through this, we confirmed significant results on the time accuracy of wireless and single-patch ECG.

Patients with chest pain were enrolled in the ED and divided into 2 groups to examine whether the timeliness with SP-ECG was superior to that with C-ECG. The results showed that timeliness was significantly higher in the intervention group, implying its usefulness in complex ED environments.

This study is the first randomized controlled trial comparing wireless and single-patch ECG, and we arrived at meaningful results. Additionally, we did not impede complex processes and workflows in the ED. It was possible to perform other tests, such as chest x-rays or laboratory tests, while the device was placed, minimizing interference with the protocols of the ED. None of the patients dropped out of this study to undergo other procedures. Moreover, none of the patients or medical staff complained that its placement interfered with the other examinations.

### Limitations

This study has some limitations. First, as this was a single-center study, the patient population was not fully representative. Therefore, our data should be validated across other institutions to draw generalizable conclusions. Second, this study was conducted over a relatively short period of time. Additionally, due to a lack of follow-up observations, the impact of the procedure in the ED could not be directly confirmed. SP-ECG has not yet been verified, and minor problems have been identified. In addition, human error occurred in 4 subjects in the SP-ECG group during the device setup and study.

### Future Directions

To prevent the error of attaching a wireless electrocardiogram to the patient inappropriately during application, the provider should receive adequate training. Additional methods to compensate for the error of attaching a wireless electrocardiogram to the patient inappropriately should be explored in the future. The use of SP-ECG is expected to reduce the demand for human resources. In this study, only time accuracy was compared and evaluated. However, it was impossible to determine the effect of improving ED problems, such as staff shortage, the complexity of the emergency room medical environment, satisfaction with the test provider, the effect on the patient's overall diagnosis, and outcome of applying SP-ECG. Therefore, further research is required [18] to fill these gaps.

### Conclusions

ECG is the most important and frequently performed test for patients with chest pain. Additionally, continuous checks, rather than one-time checks, are often required. The timing of ECG may influence patient outcomes. However, ECG recordings are sometimes delayed in the ED due to congestion and lack of human resources. To increase their timeliness, the development and use of medical devices that capture measures automatically and constantly without interfering with ED activities is required. Although our study on SP-ECG revealed that it required correction of minor device imperfections and training of medical staff before it could be used in a clinical setting, we identified significant improvement in the examination timeliness and demonstrated that it minimally disrupted the treatment processes in the ED.

## Appendix

Multimedia Appendix 1 CONSORT (Consolidated Standards of Reporting Trials) flowchart.

Multimedia Appendix 2 CONSORT-eHEALTH checklist (V 1.6.2).

## Abbreviations

C-ECG: conventional ECG
CONSORT: Consolidated Standards of Reporting Trials
ECG: electrocardiography
ED: emergency department
SP-ECG: single-patch 12-lead ECG
