# Supplementary figures and images for: Intervention in the Timeliness of Two Electrocardiography Types for Patients in the Emergency Department With Chest Pain: Randomized Controlled Trial
Source: Interact J Med Res. 2022 Sep 13;11(2):e36335. doi: 10.2196/36335 (PMC9516380; doi:10.2196/36335)

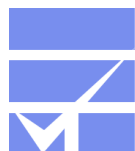

# CONSORT

TRANSPARENT REPORTING of TRIALS

## CONSORT 2010 Flow

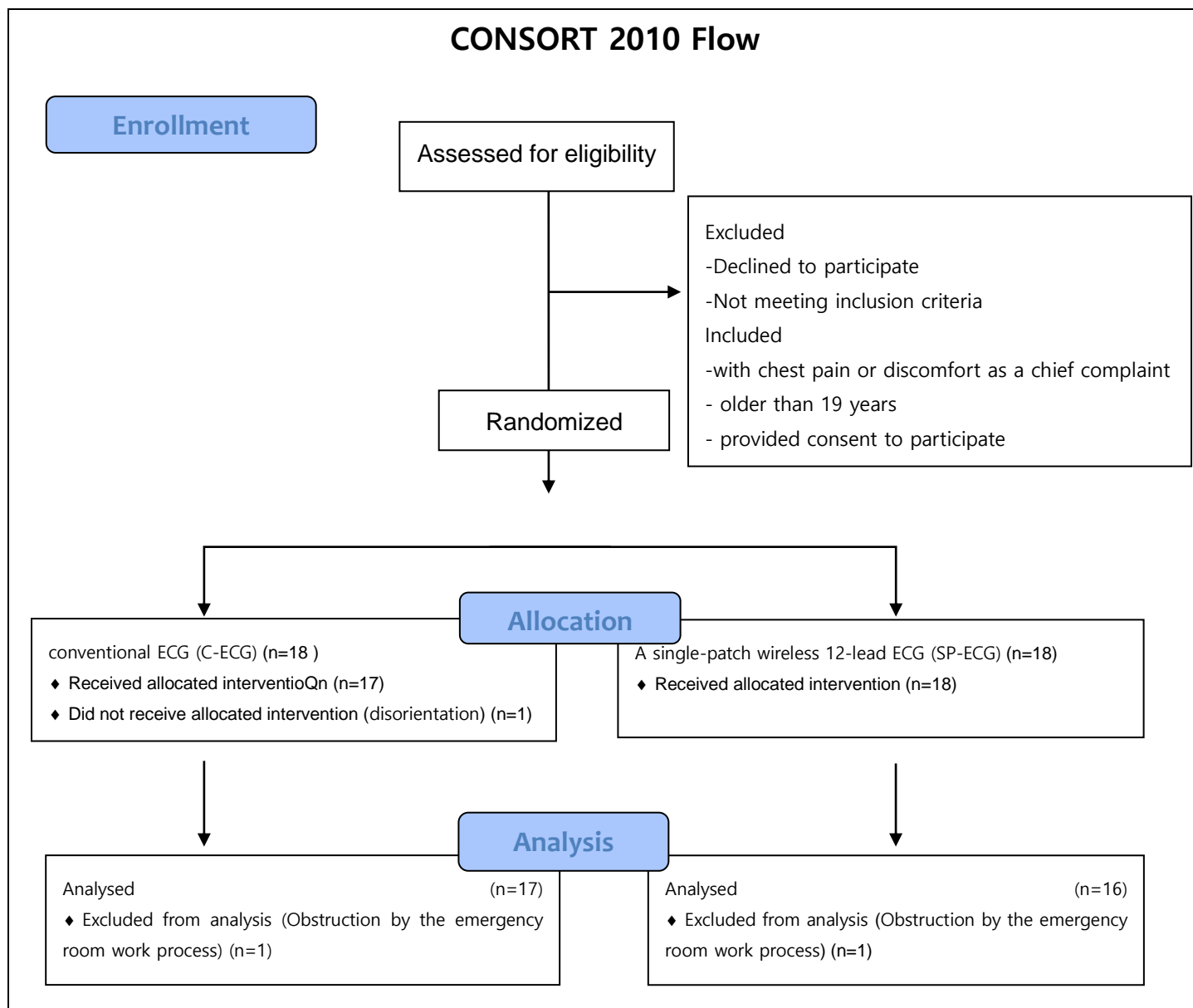

Supplement: Multimedia Appendix 1 [file ijmr_v11i2e36335_app1.pdf]
