# Supplementary material for: Intervention in the Timeliness of Two Electrocardiography Types for Patients in the Emergency Department With Chest Pain: Randomized Controlled Trial
Source: Interact J Med Res. 2022 Sep 13;11(2):e36335. doi: 10.2196/36335 (PMC9516380; doi:10.2196/36335)
Supplement: Multimedia Appendix 2 [file ijmr_v11i2e36335_app2.pdf]

CONSORT-EHEALTH Checklist V1.6.2 Report

Manuscript Number 36335

(based on CONSORT-EHEALTH V1.6), available at [http://tinyurl.com/consort-ehealth-v1-6].

Date completed
8/1/2022 21:15:41
by
suyoung YOO

Intervention in the Timeliness of Two Electrocardiography Types for Patients in the Emergency Department With Chest Pain: Randomized Controlled Trial

TITLE

1a-i) Identify the mode of delivery in the title
Intervention in the Timeliness of Two Electrocardiography Types

1a-ii) Non-web-based components or important co-interventions in title
Intervention in the Timeliness of Two Electrocardiography Types

1a-iii) Primary condition or target group in the title
for Patients in the Emergency Department With Chest Pain

ABSTRACT

1b-i) Key features/functionalities/components of the intervention and comparator in the METHODS section of the ABSTRACT
compare the time accuracy of a single-patch 12-lead ECG (SP-ECG) with that of conventional ECG (C-ECG)

1b-ii) Level of human involvement in the METHODS section of the ABSTRACT
researcher needed to support

1b-iii) Open vs. closed, web-based (self-assessment) vs. face-to-face assessments in the METHODS section of the ABSTRACT
offline, only ER visited patient

1b-iv) RESULTS section in abstract must contain use data
With a power of 0.95 and effect sizes of 0.05 and 1.36, the minimum number of samples was calculated. The minimum sample size for each SP-ECG and C-ECG group is 15.36 participants, assuming a 20% dropout rate. As a result, 36 patients with chest pain participated, and 33 of them were analyzed. The timeliness of SP-ECG and C-ECG for the first follow-up ECG was 87.5% and 47.0%, respectively (<i>P</i>=.74). It was 75.0% and 35.2% at the second follow-up, respectively (<i>P</i>=.71).

1b-v) CONCLUSIONS/DISCUSSION in abstract for negative trials
the precision of SP-ECG has not yet been proved

INTRODUCTION

2a-i) Problem and the type of system/solution
Currently, ED in South Korea is following the American Heart Association’s recommendation to take an initial ECG within 10 minutes of the patient’s visit with chest pain

2a-ii) Scientific background, rationale: What is known about the (type of) system
According to the guideline of the Journal of European Heart, ECG was performed as the first step for evaluation and treatment when patients with chest pain visited the ED

Does your paper address CONSORT subitem 2b?
A single patch would enable patients to move outside the bed and could be used in complex emergency room situations requiring many tests. However, to the best of our knowledge, no studies have been conducted on such devices in ED settings. Thus, this study aimed to evaluate the effect of SP-ECG with a timer on the timeliness of follow-up ECG in the clinical setting for patients with chest pain.

METHODS

3a) CONSORT: Description of trial design (such as parallel, factorial) including allocation ratio
This was a prospective randomized controlled study conducted in the ED of an academic tertiary hospital. Participants who visited ED with chest pain were randomly assigned into the 2 groups of conventional ECG (C-ECG) and single-patch 12-lead ECG (SP-ECG).

3b) CONSORT: Important changes to methods after trial commencement (such as eligibility criteria), with reasons
The main comparison variable was the timeliness of the recording time for the 2 ECG types.

3b-i) Bug fixes, Downtimes, Content Changes

4a) CONSORT: Eligibility criteria for participants
Patients who visited the hospital’s ED with chest pain as the chief complaint were considered for inclusion in the study

4a-i) Computer / Internet literacy

4a-ii) Open vs. closed, web-based vs. face-to-face assessments:
offline, only ER visited patient

4a-iii) Information giving during recruitment
offline, only ER visited patient

4b) CONSORT: Settings and locations where the data were collected
This study was conducted in the ED of an academic tertiary hospital in Seoul with approximately 2000 inpatient beds and around 2,000,000 annual outpatient visits. The average number of ED admissions is 78,000 per year.

4b-i) Report if outcomes were (self-)assessed through online questionnaires
no. we didn't use any online survey

4b-ii) Report how institutional affiliations are displayed
The first study participants were enrolled on July 30, 2020, while the last study participants were enrolled on October 8, 2020. The study was conducted for approximately 70 days.

5) CONSORT: Describe the interventions for each group with sufficient details to allow replication, including how and when they were actually administered

5-i) Mention names, credential, affiliations of the developers, sponsors, and owners
This study was supported by the Samsung Medical Center grant (SMO1200701).

5-ii) Describe the history/development process

5-iii) Revisions and updating

5-iv) Quality assurance methods

5-v) Ensure replicability by publishing the source code, and/or providing screenshots/screen-capture video, and/or providing flowcharts of the algorithms used

5-vi) Digital preservation

5-vii) Access
participants were not allowed to use application.

5-viii) Mode of delivery, features/functionalities/components of the intervention and comparator, and the theoretical framework
The primary outcome in this study was the timeliness of the ECG measurements. The study protocol required subjects in both groups to be subjected to an ECG twice at 15-minute intervals. Participants in the C-ECG group underwent manual measurements by medical personnel at a specified time using the same 12-lead ECG

5-ix) Describe use parameters

5-x) Clarify the level of human involvement

5-xi) Report any prompts/reminders used
Report any prompts/reminders was not used

5-xii) Describe any co-interventions (incl. training/support)
we don't use any co-interventions

6a) CONSORT: Completely defined pre-specified primary and secondary outcome measures, including how and when they were assessed
we don't use online questionnaires

**6a-i) Online questionnaires: describe if they were validated for online use and apply CHERRIES items to describe how the questionnaires were designed/deployed**		
**6a-ii) Describe whether and how “use” (including intensity of use/dosage) was defined/measured/monitored**		
**6a-iii) Describe whether, how, and when qualitative feedback from participants was obtained**		
**6b) CONSORT: Any changes to trial outcomes after the trial commenced, with reasons**		
This study was conducted in the ED of an academic tertiary hospital in Seoul with approximately 2000 inpatient beds and around 2,000,000 annual outpatient visits. The average number of ED admissions is 78,000 per year.		
**7a) CONSORT: How sample size was determined**		
**7a-i) Describe whether and how expected attrition was taken into account when calculating the sample size**		
**7b) CONSORT: When applicable, explanation of any interim analyses and stopping guidelines**		
we don't use online questionnaires		
**8a) CONSORT: Method used to generate the random allocation sequence**		
participant were randomly divided into the SP-ECG and C-ECG groups with envelop draw		
**8b) CONSORT: Type of randomisation; details of any restriction (such as blocking and block size)**		
randomization with no blocking		
**9) CONSORT: Mechanism used to implement the random allocation sequence (such as sequentially numbered containers), describing any steps taken to conceal the sequence until interventions were assigned**		
we don't have mechanism used to implement the random allocation sequence (such as sequentially numbered containers), describing any steps taken to conceal the sequence.		
**10) CONSORT: Who generated the random allocation sequence, who enrolled participants, and who assigned participants to interventions**		
one researcher generated the random allocation sequence, enrolled participants, and assigned participants to interventions .		
**11a) CONSORT: Blinding - If done, who was blinded after assignment to interventions (for example, participants, care providers, those assessing outcomes) and how**		
**11a-i) Specify who was blinded, and who wasn’t**		
not possible to blind the participants		
**11a-ii) Discuss e.g., whether participants knew which intervention was the “intervention of interest” and which one was the “comparator”**		
**11b) CONSORT: If relevant, description of the similarity of interventions**		
we don't have a placebo or sham intervention		
**12a) CONSORT: Statistical methods used to compare groups for primary and secondary outcomes**		
All data were stored in a Microsoft Excel spreadsheet. P values compared to baseline characteristics and time differences were calculated by comparing the means using the chi-square test. We compared the time differences between the 2 groups by comparing the means and standard deviations. Statistical significance was set at P<.05.		
**12a-i) Imputation techniques to deal with attrition / missing values**		
All data were stored in a Microsoft Excel spreadsheet. P values compared to baseline characteristics and time differences were calculated by comparing the means using the chi-square test. We compared the time differences between the 2 groups by comparing the means and standard deviations. Statistical significance was set at P<.05.		
**12b) CONSORT: Methods for additional analyses, such as subgroup analyses and adjusted analyses**		
All data were stored in a Microsoft Excel spreadsheet. P values compared to baseline characteristics and time differences were calculated by comparing the means using the chi-square test. We compared the time differences between the 2 groups by comparing the means and standard deviations. Statistical significance was set at P<.05.		
RESULTS		
**13a) CONSORT: For each group, the numbers of participants who were randomly assigned, received intended treatment, and were analysed for the primary outcome**		
A total of 36 participants were enrolled in this study. The median ages in the SP-ECG and C-ECG groups were 63.7 (SD 18.4) and 58.1 (SD 12.4), respectively		
**13b) CONSORT: For each group, losses and exclusions after randomisation, together with reasons**		
One of the excluded patients wanted to drop out of the study due to disorientation. In one participant from each group, there were errors in the time measurements due to study violation.		
**13b-i) Attrition diagram**		
**14a) CONSORT: Dates defining the periods of recruitment and follow-up**		
we don't have secular events		
**14a-i) Indicate if critical “secular events” fell into the study period**		
**14b) CONSORT: Why the trial ended or was stopped (early)**		
the trial not ended or was not stopped early.		
**15) CONSORT: A table showing baseline demographic and clinical characteristics for each group**		
we don't have any ehealth trials		
**15-i) Report demographics associated with digital divide issues**		
we don't have any ehealth trials		
**16a) CONSORT: For each group, number of participants (denominator) included in each analysis and whether the analysis was by original assigned groups**		
**16-i) Report multiple “denominators” and provide definitions**		
The average age of the 33 final participants was 61.06 (SD 15.8) years. Moreover, 14/33 (42.4%) patients were women, and the most common Korean Triage and Acuity Scale was 3. The other characteristics did not show significant intergroup differences		
**16-ii) Primary analysis should be intent-to-treat**		
**17a) CONSORT: For each primary and secondary outcome, results for each group, and the estimated effect size and its precision (such as 95% confidence interval)**		
For the first follow-up ECG, the timeliness values of the recordings in the SP-ECG and C-ECG groups were 13/16 (81%) patients and 7/17 (41%) patients, respectively (P=.74). At the second follow-up, it was 10/16 (63%) patients and 6/17 (35%) patients, respectively (P=.71). Overall, the accuracies were 81.2% and 41.1%, respectively (P=.62).		
**17a-i) Presentation of process outcomes such as metrics of use and intensity of use**		
**17b) CONSORT: For binary outcomes, presentation of both absolute and relative effect sizes is recommended**		
For the first follow-up ECG, the timeliness values of the recordings in the SP-ECG and C-ECG groups were 13/16 (81%) patients and 7/17 (41%) patients, respectively (P=.74). At the second follow-up, it was 10/16 (63%) patients and 6/17 (35%) patients, respectively (P=.71). Overall, the accuracies were 81.2% and 41.1%, respectively (P=.62).		
**18) CONSORT: Results of any other analyses performed, including subgroup analyses and adjusted analyses, distinguishing pre-specified from exploratory**		
For the first follow-up ECG, the timeliness values of the recordings in the SP-ECG and C-ECG groups were 13/16 (81%) patients and 7/17 (41%) patients, respectively (P=.74). At the second follow-up, it was 10/16 (63%) patients and 6/17 (35%) patients, respectively (P=.71). Overall, the accuracies were 81.2% and 41.1%, respectively (P=.62).		
**18-i) Subgroup analysis of comparing only users**		
**19) CONSORT: All important harms or unintended effects in each group**		
we don't have important harms or unintended effects		
**19-i) Include privacy breaches, technical problems**		
**19-ii) Include qualitative feedback from participants or observations from staff/researchers**		
DISCUSSION		
**20) CONSORT: Trial limitations, addressing sources of potential bias, imprecision, multiplicity of analyses**		
**20-i) Typical limitations in ehealth trials**		

The C-ECG group consisted of 6/17 (35%) women and 11/17 (65%) men, whereas the SP-ECG group consisted of 8/16 (50%) women and 8/16 (50%) men. In the C-ECG group, the average time of the first follow-up ECG in women (n=6) was 22 (SD 6.35) minutes, while that of the second follow-up ECG was 82.5 (SD 55.20) minutes. For men (n=11), the average time of the first follow-up ECG was 22 (SD 12.55) minutes, while that of the second ECG was 60 (SD 66.28) minutes. In the SP-ECG group, the average time for women's (n=8) first follow-up ECG was 15 (SD 1) minutes, while that of the second ECG was 32 (SD 3.98) minutes. For men in the SP-ECG group (n=8), the average time for the first follow-up ECG was 17 (SD 4.58) minutes, while that for the second follow-up ECG was 32 (SD 4.71) minutes.		
**21) CONSORT: Generalisability (external validity, applicability) of the trial findings**		
**21-i) Generalizability to other populations**		
**21-ii) Discuss if there were elements in the RCT that would be different in a routine application setting**		
**22) CONSORT: Interpretation consistent with results, balancing benefits and harms, and considering other relevant evidence**		
**22-i) Restate study questions and summarize the answers suggested by the data, starting with primary outcomes and process outcomes (use)**		
The C-ECG group consisted of 6/17 (35%) women and 11/17 (65%) men, whereas the SP-ECG group consisted of 8/16 (50%) women and 8/16 (50%) men. In the C-ECG group, the average time of the first follow-up ECG in women (n=6) was 22 (SD 6.35) minutes, while that of the second follow-up ECG was 82.5 (SD 55.20) minutes. For men (n=11), the average time of the first follow-up ECG was 22 (SD 12.55) minutes, while that of the second ECG was 60 (SD 66.28) minutes. In the SP-ECG group, the average time for women's (n=8) first follow-up ECG was 15 (SD 1) minutes, while that of the second ECG was 32 (SD 3.98) minutes. For men in the SP-ECG group (n=8), the average time for the first follow-up ECG was 17 (SD 4.58) minutes, while that for the second follow-up ECG was 32 (SD 4.71) minutes.		
**22-ii) Highlight unanswered new questions, suggest future research**		
Other information		
**23) CONSORT: Registration number and name of trial registry**		
we don't have Registration number and name of trial registry		
**24) CONSORT: Where the full trial protocol can be accessed, if available**		
full trial protocol can be accessed in clinicaltrials.gov		
**25) CONSORT: Sources of funding and other support (such as supply of drugs), role of funders**		
This study was supported by the Samsung Medical Center grant (SMO1200701).		
**X26-i) Comment on ethics committee approval**		
The protocol of this study was reviewed and approved by Samsung Hospital's Institutional Review Board (IRB #2019-01-046-008).		
**x26-ii) Outline informed consent procedures**		
**X26-iii) Safety and security procedures**		
**X27-i) State the relation of the study team towards the system being evaluated**		
